# Population Migration: Implications for Lymphatic Filariasis Elimination Programmes

**DOI:** 10.1371/journal.pntd.0002079

**Published:** 2013-03-28

**Authors:** K. D. Ramaiah

**Affiliations:** Vector Control Research Centre (ICMR), Medical Complex, Indira Nagar, Pondicherry, India; University of Notre Dame, United States of America

## Abstract

Human population migration is a common phenomenon in developing countries. Four categories of migration—endemic to nonendemic areas, rural to urban areas, non-MDA areas to areas that achieved lymphatic filariasis (LF) control/elimination, and across borders—are relevant to LF elimination efforts. In many situations, migrants from endemic areas may not be able to establish active transmission foci and cause infection in local people in known nonendemic areas or countries. Urban areas are at risk of a steady inflow of LF-infected people from rural areas, necessitating prolonged intervention measures or leading to a prolonged “residual microfilaraemia phase.” Migration-facilitated reestablishment of transmission in areas that achieved significant control or elimination of LF appears to be difficult, but such risk can not be excluded, particularly in areas with efficient vector-parasite combination. Transborder migration poses significant problems in some countries. Listing of destinations, in endemic and nonendemic regions/countries, and formulation of guidelines for monitoring the settlements and the infection status of migrants can strengthen the LF elimination efforts.

## Introduction

A global programme to eliminate lymphatic filariasis (LF) was launched in the year 2000. Close to 1.4 billion people living in 72 countries are targeted for annual mass drug administration (MDA), the strategy for eliminating LF (http://www.who.int/lymphatic_filariasis/disease/en/). Five to six rounds of MDA has the potential to eliminate LF in a significant proportion of communities [1]–[5]. The national LF elimination programmes of some countries completed administration of 5–6 MDAs to the entire endemic population. Some countries completed the MDA in a proportion of endemic provinces. With the advances made by the MDA programmes in many countries, the need arises to understand the importance of various human factors that pose threats to the gains of the programmes. Human migration is one such factor. Migration of people in search of employment has been a common and increasing phenomenon in developing countries. Both permanent or long-term and temporary migrations are common in many communities. Information is scarce on the role of migrants in creation of new foci and/or reintroduction of transmission into the “cleansed” areas, which are potential threats to LF elimination efforts. Also, human migration had been identified as an important determinant of the success of LF elimination programmes [6]. Available information has been collected in this paper to promote understanding of the importance and implication of migration for LF elimination programmes.

Four categories of migration have implications for LF elimination efforts. These are (i) migration from endemic areas to nonendemic areas, (ii) migration from endemic rural areas to endemic urban areas, (iii) migration from endemic areas to the areas that achieved control/elimination of LF, and (iv) transborder migration.

## Migration from Endemic Areas to Nonendemic Areas

Millions of people from endemic areas of the endemic countries migrate to nonendemic areas within or outside their countries. A considerable proportion of them are semiskilled workers who migrate from LF-endemic countries to countries in the Persian Gulf region, Southeast Asia, Europe, and North America. LF has been one of the commonest infections among them.

Persian Gulf countries are the home and destination for millions of workers from South Asian and Southeast Asian countries, a major LF-endemic region. An investigation among immigrant workers in Kuwait showed LF antigenaemia (Ag) prevalence of 18%–20% and microfilaria (Mf) prevalence of 3.0%. Ag was detected in 2 of the 260 local residents, suggesting very low levels of transmission [7]. An Mf rate of 3.5% was reported in immigrants in Saudi Arabia, who came from five Southeast Asian countries [8]. Often, the local culicine species, *Culex pipiens pipiens* and *C. quinquefasciatus*, were found to be susceptible to infection with *Wuchereria bancrofti* [8], [9]. However, the unfavourable hot environmental conditions for the survival and propagation of the vectors and the economic prosperity of the Persian Gulf countries make autochthonous transmission and establishment of active transmission foci difficult. While low levels of *W*. *bancrofti* infection were observed among Myanmar migrants in Bangkok, Thailand, no infection was found in local people [10], although the local strain of *C*. *quinquefasciatus* was found to be susceptible to infection from migrants [11].

Nearly a quarter of the immigrants examined in hospitals in Spain were found infected with LF [12], [13]. Many European countries and the United States are the prominent destinations and homes for a significant number of immigrants from developing countries. New Zealand is the destination for migrants from the Cook Islands and other island nations in the South Pacific region, endemic for *Aedes*-transmitted diurnally subperiodic *W. bancrofti* [14], [15]. While the mosquito-free environment or negligible man-vector contact situations of these developed countries prevents the spread of infection from the migrant population, health care personnel need to be prepared to diagnose and treat clinical symptoms among the migrants [16]. This is also important from the perspective of prevention and management of chronic LF, a major goal of LF elimination programmes.

Migration also occurs from endemic to nonendemic regions within a country. For example, in India, some towns and cities in the nonendemic northern-most and northwestern parts of the country have a long history of population influx from many endemic states. Up to 6% of this population was found to harbour Mf. However, none of the local residents were found with Mf [17]–[19]. A similar situation was observed in Little Andaman island, where 3 out of the 12 surveyed villages were found to have people infected with nocturnally periodic *W. bancrofti* infection. The Mf rate in the 3 villages was in the range of 1.02%–6.45%. All the infected people were found to be immigrants from endemic parts of India and Bangladesh. Although 0.24% of the 442 dissected *Cx. quinquefasciatus* were found to harbour infection and one mosquito was found with infective stage larvae, none of the local residents were found with microfilaraemia [20].While such migration is common in many endemic countries, published information is very scarce.

Identification of settlements with inward migrants within the countries and provinces, regular screening of the immigrant workers and neighbourhood households, and treatment of infected people will protect the infected immigrants from developing chronic disease condition and the local community from risk of exposure to infection. Some guidelines from WHO on monitoring of LF infection in immigrants for all the destination countries may be useful.

## Migration from Endemic Rural Areas to Urban Areas

A few decades ago, LF and its major vector, *C. quinquefasciatus*, were predominantly urban problems and the dispersal of LF was from urban to rural areas [21]. While a few studies suggest that LF has been declining in many urban population groups [22], [23] due to improving socioeconomic conditions and household-level vector control measures, the problem in rural areas had been assuming serious proportion. Rural areas have been witnessing an increase in the density of *C. quinquefasciatus* as a result of ecological changes (ex., increased piped water supply, construction of drainage canals and their poor maintenance). Thus, rural populations, in the absence of intervention measures, are at higher risk of LF infection and many communities were found with very high prevalence rates. Hence, rural–urban migration is an important epidemiological factor.

Information on some aspects of migration was collected in 2004–2005, through unstructured interviews, in 20 endemic villages in the Villupuram district of Tamil Nadu, South India, where longitudinal studies were implemented to assess and compare the impact of mass treatment with DEC alone, Ivermectin alone, DEC+Ivermectin, and DEC+Albendazole [3], [24], [25]. The information reveals that rural to urban migrants account for 90% of the total migrants. Over a period of 13 years, about 12% of the families migrated. Nearly 23% of the population migrated, either temporarily or permanently. The proportion of males and females among migrants is nearly equal. Their destinations include 43 urban or rural areas. The most preferred destination of the migrants was the nearest city, Chennai, a well-known industrial hub located 150 km away, and with a population of >5 million. About 41% of the migrants preferred the city and 19% the nearby towns, located 40–60 km away. Eighteen percent left the villages upon getting married. Seventy-six percent of the migrants are from among the most productive age-groups of 11–40 years. These age-groups are also known to have a higher proportion of Mf carriers [26]. Another study in India showed that within a span of one year, 17%–27% of the population moved out of the villages during different seasons [27]. On average, 2.7% of the population moved out of the endemic villages every year in Papua New Guinea [28].

The pre-MDA Mf rate among the migrant population from some endemic rural areas ranged from 17.0%–39.0% [24]–[26]. These data suggest that there could be a steady inflow of a considerable number of people with LF infection from non-MDA rural areas to their destinations, which are mostly towns and cities in many countries. The available Mf prevalence data, collected at a yearly interval to evaluate the impact of MDA [24]–[26], show that the people who migrated from 20 villages after receiving a few rounds of MDA harbour much less Mf prevalence than those who migrated prior to MDA, and 0.0%–5.0% of those migrated after 1–8 rounds of MDA were found to harbour Mf. Some of them could be residual Mf carriers. In the countries where the rural and urban filariasis is transmitted by the same vector, the rural migrants constitute an important source of infection in urban areas. Even when the urban vectors are different from rural vectors, transmission potential of the former cannot be under estimated [28]. Rural–urban migration assumes significance when there is a potential for establishment of an urban transmission cycle, as was seen in some urban areas of Ghana [29].

Most of the migrants are likely to settle either at work sites in urban areas or in the localities where socioeconomically poorer segments of the population live. These groups were shown to have higher Mf prevalence than the more prosperous socioeconomic groups [23]. However, the former are more willing to participate in MDA programmes [30]. Thus, the migrants to urban areas do have fair chances of undergoing MDA, if it is in place, and getting rid of LF infection, and they may not pose a “special risk” to the LF elimination programmes. However, in view of the poorer performance of the MDA programmes in urban areas [30], [31], its strengthening, particularly in poorer localities, is necessary to effectively control LF in poorer urban sections as well as in the migrant population.

The declining trend of LF in some urban areas such as Cochin [22] and Chennai [23] in India, into which migration is also rampant, indicates that the “urban factors”—use of personal protection against mosquitoes and improving housing and health care—may inhibit to some extent the full potential of the migrants to contribute to transmission. These urban factors include use of personal protection measures against mosquitoes by ≥75% of households [23],[32],[33], improvement of housing due to affordable housing programmes [34], higher economic growth in developing countries, and better affordability of medicines and health care. However, the migrants can prolong the “residual microfilaraemia” phase of the LF infection, necessitating prolonged intervention and monitoring.

## Migration from Endemic or Non-MDA Areas to Areas that Achieved Control/Elimination of LF

In many endemic countries, simultaneous implementation of MDA in all endemic provinces and rural and urban areas may be logistically difficult. In such situations, the provinces or areas that start MDA earliest may achieve first significant control or elimination of LF. These areas are vulnerable to the threat of introduction of infection through migrants from non-MDA endemic areas, in addition to the threat of resurgence of infection from local residual Mf carriers. The chances and pace of reestablishment of transmission in such areas may primarily depend upon the ability of local vectors. The ability of *Aedes* vectors to transmit LF is relatively higher than *Culex* and *Anopheles* vector species [35]. Therefore, infected migrants may pose more of a threat in *Aedes* vector areas. Historically, these areas are also known to be prone to resurgence of infection from low endemicity levels. In some island countries in the South Pacific region, where *Aedes* species are the vectors, resurgence of LF infection was observed from very low levels of postintervention LF infection [36], [37]. And interisland migration is a common phenomenon in the region, although the role of migrants in the resurgence of infection remains underinvestigated. Some evidence is available to suggest that reintroduction of infection into the areas cleared of LF is difficult in *Anopheles* vector areas [38]. Although the data on inward migration is lacking, in Trinidad, where *C. quinquefasciatus* was the vector, no reintroduction of LF was observed over a 21-year follow-up study [39], [40]. Thus, migrants may not pose a problem in successful areas where *Culex* and *Anopheles* species are the vectors. However, effective surveillance that includes monitoring the migration pattern and infection status of migrants is essential in successful areas where *Aedes* species are the vectors. The challenges of surveillance in such areas were discussed by Huppatz et al. [15].

## Transborder Migration

One of the most well-known examples of the LF threat posed by transborder migration is the Thailand-Myanmar border. The border areas were endemic for LF and pose a challenge to LF elimination efforts in Thailand. An Mf rate up to 6.0%, Ag prevalence in the range of 22.0%–36.8%, and antibody prevalence of 54% was reported in border areas within Thailand [41], [42]. The efforts to eliminate LF in the border areas are confounded by the influx of migrants from Myanmar. The Mf rate among the Myanmar migrants ranged from 4.4%–8.0%, Ag prevalence 10.0%–24%, and an antibody prevalence of 42% was reported [43], [44]. While the information on transborder migration is scarce, it appears to be more important than hitherto thought and poses a problem in Southeast Asia, Africa, and South Pacific regions. For example, information on the risk posed by migrants from neighbouring countries that have implemented effective MDA programmes (Thailand, Cambodia) and/or reduced LF infection significantly (Cambodia, Togo, Solomon Islands, etc.) is not clear. In the areas where it poses problem, concerted efforts by the programmes on either side of the border are important. Identification of such transborder risk areas and joint implementation of an LF elimination programme and intensive monitoring and evaluation may be necessary.

## Operational Research Needs

- Identify the most important destination towns and cities, in each province of the endemic countries, prone to inward migration, evaluate the LF infection levels of migrants and the local population during the course of MDA, and assess if such areas require any special efforts to eliminate LF.
- Develop guidelines for known nonendemic countries for periodical evaluation of the LF risk posed by the immigrant population, and treatments for infected people.

## Conclusions

Information on the implications of migration to LF control/elimination is scanty. Available evidence suggests that there is a considerable dispersal of LF-infected people from endemic areas to nonendemic areas and within endemic areas. The implications of migration to LF elimination efforts appear to be more serious (i) in urban areas due to the predominant rural–urban migration and (ii) in areas with efficient vector-parasite combination and/or areas with a history of infection resurgence. No evidence is available in the recent past to suggest that migrants pose a threat in establishment of active transmission foci and causing new infections in known nonendemic areas and in the non-*Aedes* vector areas, where LF elimination has been achieved. This is particularly so in the present era of increasing use of personal protection measures and improvement in housing conditions. Nevertheless, in light of the ambitious goals of the global programme to eliminate LF, it is necessary and safe to establish sentinel sites and periodical monitoring and evaluation mechanisms to assess LF infection levels in (i) important destinations in nonendemic areas of endemic countries and nonendemic countries, (ii) areas that achieved LF elimination, and (iii) important destinations within urban areas.

**Key Learning Points**

- Annually in some regions, about 2.0%–2.5% of the endemic rural population migrates, raising the risk of introduction of infection or prolonging the “residual infection” phase in their predominant destinations of towns and cities.
- Migrants have the potential to thwart the achievements of the control/elimination programmes in areas with efficient vector-parasite combination and transborder areas.
- Guidelines are required to monitor the migrant-prone areas and alleviate the impact of migration.

**Key Papers in the Field**

- Alexander ND, Bockarie MJ, Dimber ZB, Griffin L, Kazura JW, et al. (2001) Migration and dispersal of lymphatic filariasis in Papua New Guinea. Trans R Soc Trop Med Hyg 95: 277–279.
- Gbakima AA, Appawu MA, Dadzie S, Karikari C, Sackey SO, et al. (2005) Lymphatic filariasis in Ghana: establishing the potential for an urban cycle of transmission. Trop Med Int Health 10: 387–392.
- Huppatz C, Durrheim D, Lammie P, Kelly P, Melrose W (2008) Eliminating lymphatic filariasis–the surveillance challenge. Trop Med Int Health 13: 292–294.
- Sunish IP, Rajendran R, Mani TR, Gajanana A, Reuben R, et al. (2003) Long-term population migration: an important aspect to be considered during mass drug administration for elimination of lymphatic filariasis. Trop Med Int Health 8: 316–321.
- Triteeraprapab S, Songtrus J (2001) High prevalence of *Wuchereria bancrofti* infection among Myanmar migrants in Thailand. Ann Trop Med Parasitol 95: 535–538.
